# Training professionalism trainers

**Published:** 2018-09-09

**Authors:** Fateme Alipour, Fariba Asghari, Homayoun Amini

**Affiliations:** 1 *Associate Professor, Eye Research Center, Farabi Eye Hospital, Tehran University of Medical Sciences, Tehran, Iran.*; 2 *Associate Professor, Medical Ethics and History of Medicine Research Center, Tehran University of Medical Sciences, Tehran, Iran.*; 3 *Professor, Department of Psychiatry, Roozbeh Psychiatric Hospital, Tehran University of Medical Sciences, Tehran, Iran.*

***What problems were addressed*** *?* ***Abstra***

*io* *n* *alism, Undergraduate medical student, Content analysis, In-depth interview*

 Informal learning through interaction between a teacher and a learner could not be replaced or compensated by formal education (1). In fact, if attending physicians do not believe in the spirit of professionalism, or do not model professional behavior, could have a reverse effect on trainers’ attitudes. Integrating and enforcing professionalism besides other scientific and technical skills, needs all clinical attending staff be familiar with its concept and theoretical basics, and actively consider it in their own behaviors and dialogues. The prerequisite of teaching professionalism to clinical residents –who are challenged more in busy clinical setting- needs ensuring that all attendings are potent enough. This innovation was designed to train enough professionalism trainers of clinical staff.

***What was tried?***

A core committee of 12 clinical staff and ethicists was developed. All of these professors had previously attended the faculty development workshops in the field of professionalism. Each member chose at least one topic out of the list of defined topics: the concept of professionalism, excellence, justice, responsibility, altruism, respect, autonomy, honor and integrity, conflict of interest, and ethical reasoning, and prepared for presenting it in peer-review sessions which was held monthly. After each peer-review session, feedback was provided on the content, teaching method, and teaching materials used by the presenter by all participants. Concurrently, faculty development workshops were held in Educational Developmental Center (EDC) of the Tehran University of Medical Sciences (4 times a year) by these empowered trainers. After each workshop, it was announced that any interested professors were welcomed to join the group; so the group was actively expanding. So far, 14 peer-review sessions attended by 42 participants with a combination of faculty members from the Ethics and Clinical Departments have been held to peer-review and provide feedback on the sessions. Finally, 16 new trainers in 25 person/topics have been prepared for teaching at the workshops for the empowerment of clinical professors in the context of professionalism. This team has held ten workshops at the EDC and 8 workshops in 6 subsidiary hospitals of the university, and has empowered a total of more than 300 faculty members in the professionalism and its related topics.

***What lessons were learned?***

With regard to the skills and capabilities of the medical practice, such as professional commitment and communication skills, impact of lessons provided by clinical trainers -familiar with real challenges-, is higher. So, potent trainers can be trained using the peer-review sessions with active participation of a team consisted of both clinical disciplines and ethicists. This method combines the advantage of being familiar with real challenges and enough self-confidence of the clinical trainers.
